# Skin-derived volatile organic compounds trigger redox signalling pathways in human keratinocytes *via* gas-phase interaction

**DOI:** 10.1039/d5ra02839f

**Published:** 2025-09-10

**Authors:** M. Finnegan, V. Bolikava, N. Walsh, A. Morrin

**Affiliations:** a School of Chemical Sciences, Insight Research Ireland Centre for Data Analytics, The RAPID Institute, Dublin City University D09 V209 Ireland aoife.morrin@dcu.ie; b School of Biotechnology, Life Sciences Institute, Dublin City University D09 V209 Ireland

## Abstract

Human skin emits a diverse range of volatile organic compounds (VOCs) originating from both endogenous metabolic activity and microbial transformation of sweat and sebum. While these volatiles have been profiled extensively, their potential to influence host cellular processes remains largely unexplored. In this study, we investigate the capacity of five skin-relevant VOCs—nonanal, decanal, 6-methyl-5-hepten-2-one (6MHO), acetic acid (AA), and 2-ethyl-1-hexanol (2EH) – to induce redox signalling pathways in keratinocytes. We demonstrate that selected compounds, particularly nonanal, decanal and AA, induce intracellular reactive oxygen species (ROS) and activate the Nrf2–Keap1 antioxidant defence mechanism. Using both conventional liquid-phase treatment and a custom-designed headspace system for gas-phase treatment, we show that these VOCs elicit this signalling response from both liquid and gas phases. These findings provide the first mechanistic evidence that endogenous or microbially-derived VOCs from skin can function as gaseous redox modulators, capable of triggering protective cellular responses from a distance. This work presents new evidence for cell–cell volatile communication in skin and through its resident microbiota, offering insights into the signalling potential of volatile metabolites.

## Abstract 2

Skin-derived volatile organic compounds in both liquid and gas phases trigger cellular responses in human keratinocytes. Certain VOCs induce reactive oxygen species production and activity the Nrf2-Keap1 antioxidant pathway.

## Graphical abstract

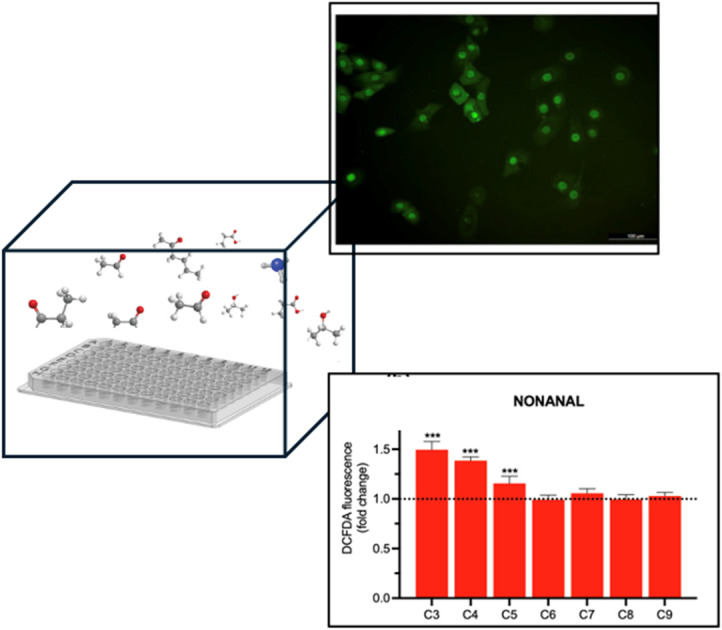

## Introduction

Human skin emits a diverse volatile organic compound (VOC) profile comprising small compounds and metabolites produced from endogenous gland secretions as well as exogeneous metabolites derived from the skin microbiota and from their metabolization and transformation of gland sweat and sebum secretions.^1,2^ Volatiles emitted from skin include a variety of compound classes such as alkanes, alkenes, aldehydes, acids, ketones, alcohols, and sulphur- and nitrogen-containing compounds.^3^ A range of amines are also be produced, as well as smaller signalling molecules such as hydrogen sulphide and nitric oxide.^4^

Many of the acids, alcohols and aldehydes reported to be in the skin volatile emission originate from the interactions between sebaceous gland secretions and cutaneous bacteria.^5^ Skin is inhabited by a wide variety of microbes including diverse bacterial species which colonize different regions of the skin, depending on conditions.^6,7^ Anaerobic bacteria living in the hair follicle/sebaceous gland duct use lipases to liberate long-chain acids from triglycerides, which are further metabolized by aerobic bacteria into longer, saturated and unsaturated acids and smaller volatile acids, aldehydes and alcohols.^8^ Acetic acid (AA), for example, is commonly recovered from skin and considered to be predominantly produced microbially, supported by several *in vitro* culture studies including that from skin-resident bacteria including *S. aureus* and *S. epidermidis*.

Another significant source of some of these smaller volatile compounds in skin is from oxidative stress-initiated processes. Oxidative stress results in the oxidation of polyunsaturated fatty acids in cell membrane lipid bilayers for example, leading to the formation and accumulation of unsaturated aldehydes. Nonanal and decanal for example are frequently recovered from skin and are likely to, at least in part, be derived from oxidative stress-linked cellular processes. Both nonanal and decanal, as well as other volatile aldehydes have been found to be elevated in skin with ageing^9^ and disease progression,^10^ supporting their links to oxidative stress.

Atmospheric chemistry can also play an exogenous role in triggering oxidative stress and the production of VOCs at the skin surface. For example, ozone reacts with squalene, a constituent of skin lipids, to produce compounds containing carbonyl, carboxyl or a-hydroxyketone functional groups. Prominent compounds recovered from skin as a result of ozonolysis include 6-methyl-5-hepten-2-one (6MHO) and geranylacetone.^11^ Although low molecular weight alcohols are commonly seen in primary microbial metabolism, the larger alcohol, 2-ethyl-1-hexanol (2EH) has also been recovered from skin previously,^9^ although its source is not yet elucidated. One hypothesis is that it is linked to the hydrolysis of plasticizers present in indoor air atmospheres and cosmetics products applied to skin, and could be produced through enzymatic hydrolysis or other degradation processes potentially at the skin surface.^12^

Oxidative stress, which is thought to lead to the production of certain VOCs, can be triggered endogenously (*e.g.* mitochondrial peroxidase activity and metabolism can also trigger the cellular production of reactive oxygen species (ROS) as well as exogenously (UV radiation, atmospheric pollution, *etc.*). The ROS species that are produced then activate the antioxidant defence system and promote the expression of antioxidant enzymes through the transcription factor nuclear factor erythroid 2 (Nrf2), one of the key regulators, to reduce ROS production. When the antioxidant defence system fails to completely scavenge ROS, oxidative stress is induced causing outcomes including lipid peroxidation and damage to DNA and proteins. Low level ROS production is important and beneficial for a wide range of physiological processes; and redox homeostasis is reached when the beneficial and harmful effects of ROS are balanced. Living organisms have evolved their cellular defence mechanisms against oxidative stress to maintain redox homeostasis.

The Nrf2-related factor-2-kelch-like ECH-associated protein 1 (Nrf2-Keap1) pathway is a major cellular defence mechanism to modulate oxidative stress.^13^ Found in the cytoplasm, the Nrf2 transcription factor is synthesized and regulated by multiple factors including the metalloprotein Keap1. When an electrophile is introduced, this triggers the oxidation of cysteine residues on the Keap1, thus inhibiting the Keap1 proteasomal degradation of Nrf2 which can then stabilize and translocate to the nucleus.^14^ After accumulation in the nucleus, Nrf2 heterodimerizes with small musculoaponeurotic fibrosarcoma proteins (Maf) and binds to antioxidant response elements (ARE) for the robust induction of cytoprotective genes for enzymes involved in the detoxication of ROS and other oxidants.^15^

Activation of this Nrf2-Keap1 signalling pathway is achieved by either direct or indirect activation. Direct activation is where an electrophile binds directly to Keap1 as described above.^16^ It has previously been shown that the α,β-unsaturated aldehydes are direct pathway activators *via* direct oxidation of Keap1. 4-Hydroxynonenal (HNE) for example is an advanced lipid peroxidation end product (ALE) and common skin metabolite, which has been shown to directly activate the Nrf2-Keap1 pathway, acting as a Michael acceptor, by changing the conformation of Keap1.^17,18^ Indirect activation can also occur by activation of an indirect antioxidant system whereby increased ROS production in cells triggers the pathway.^18–20^ Direct activation has been shown to be oxygen-independent while the indirect mechanism of activation is ROS-dependent and requires constant activation by endogenously produced compounds of this mechanism. Indirect activation is beneficial and helps to protect against significant oxidative stress events. This has led to the skin being described as having a hormetic mechanism, where low doses of oxidative stress driving sources induces an adaptive beneficial effect in cells whereas high doses cause cellular damage and death.^21–23^ Recent research has shown that treatment of keratinocyte cells with the unsaturated aldehydes nonanal and decanal as well as the aromatic aldehyde, benzaldehyde, trigger Nrf2-Keap1 activation through an indirect, ROS-dependant mechanism.^21^ In this research, a significant increase in mitochondrial membrane potential was noted in cells when treated with the aldehydes, indicating ROS augmentation. This work is of particular interest as these aldehydes are derived from skin and its microbiota and thus indicate the potential importance of volatile metabolites from skin microbiota as well as cellular processes as positive feedback inducers of the protective mechanism in skin. While such studies provide an insight into triggering cell-signalling pathways directly from the liquid phase, they do not directly show evidence of cell-signalling that can be triggered by such compounds *via* the volatile phase.^22–24^

Only very few studies have demonstrated cell signalling by metabolites directly from the volatile phase to show the potential role of VOCs as signalling or communication agents that can exchange with neighbouring cells. One example from these studies was the use of a specific headspace to study the impact on changes in cell growth of VOCs emitted from monocultures as well as from co-cultures of cancer cells without physical contact.^25^ In another example, a bronchiole organoid was used to study the immune response to microbial volatile compounds produced by *P. aeruginosa* metabolism.^26^ Although the study of VOCs as signalling agents is exciting and cutting edge, it can be challenging to study given the many unknowns and various complexities.

We hypothesize that VOCs originating from host tissue and skin-resident microbiota may function as short-range chemical messengers, capable of modulating cellular signalling pathways in neighbouring epidermal cells. In particular, we propose that certain VOCs can activate the Nrf2–Keap1 antioxidant defence mechanism in human keratinocytes through mechanisms linked to ROS generation. To test this, we selected a small panel of VOCs frequently recovered in high abundances from human skin using our methods^9^ – nonanal, decanal, 6MHO, AA and 2EH – representing both endogenous metabolic and exogenous environmental origins as well as spanning a range of physicochemical properties including volatility and lipophilicity and compound class. Using both conventional aqueous-phase exposure and a custom-designed enclosed headspace (HS) system to enable controlled volatile phase interactions, we investigated the capacity of each of these compounds to trigger oxidative stress responses and downstream Nrf2 activation. This study aims to provide mechanistic insight into the role of skin-emitted volatiles in epidermal cell signalling and redox homeostasis.

## Materials & methods

### Cell lines and culture conditions

Normal human epidermal keratinocytes (adult NHEKs) (Passage 2) (Lonza Bioscience) were cultured in medium comprised of KBM™ Gold Basal Medium (00192151) and KGM™ Gold SingleQuots supplements (Lonza). Cultures were incubated in 5% CO_2_ at 37 °C. Upon arrival, cells were thawed in a water bath at 37 °C and seeded in a T75 cm^3^ flask and allowed to grow for 5 days to ensure 70–80% confluency. Cells were then sub-cultured (1 : 3) once 70–80% confluency was reached. Cells at passage 3 or 4 were used for all experiments and cell media changed every 2–3 days. Cell morphology was examined using a light microscope and images taken at 10× magnification.

### Cell cytotoxicity assay

Cells were trypsinized as per ReagentPack protocol (Lonza). 100 μL of a 3 × 10^4^ cells per mL solution was seeded in 36 wells of 96-well plates (Greiner CELLSTAR clear 96-well plate, Merck, Ireland). After 24 h, the wells were treated with 2× concentrations of nonanal, decanal, 6MHO, AA or 2EH (Merck, Ireland) in 100 μL media. To prepare standard concentrations of the compounds, 2 M stock solutions in dimethyl sulfoxide (DMSO) (Merck, Ireland) of each compound was prepared and concentrations ranging from 0.0007–7000 μM prepared by serial dilution in cell media (*n* = 4 for each concentration). As a control, cells were also treated with DMSO whereby the final concentration of DMSO was 0.7%. Seeded cells were exposed to all compounds at different concentrations for 72 h in 5% CO_2_ at 37 °C. Cell viability reagent, PrestoBlue (ThermoFisher Scientific, Ireland), was then added (20 μL) and incubated for a further 5 h in 5% CO_2_ at 37 °C. A Tecan Infinite F200 microplate reader (top reading mode; excitation wavelength of 560 nm; emission wavelength of 590 nm), was used to read fluorescence. Background fluorescence was calculated using a blank consisting of cell media only. % viability was calculated relative to untreated controls (cells and media).

### Reactive oxygen species (ROS) assay

Cells were first trypsinized and 100 μL of a 3 × 10^4^ cells per mL solution was seeded into 40 wells of a black-walled clear bottom 96-well plate (Corning clear bottom black 96-well plate, Fisher Scientific, Ireland). Cells were then incubated for 24 h in 5% CO_2_ at 37 °C.

#### Liquid phase

Following incubation, media was aspirated from wells and cells were simultaneously treated with 100 μL of varying 1× concentrations of nonanal, decanal, 6MHO, AA or 2EH prepared in cell media, together with 100 μL dichlorofluorescein diacetate (DCFDA)-based ROS detection agent (ROS-ID Total ROS detection kit, ENZO Life Sciences) for both 2 h and 24 h in 5% CO_2_ at 37 °C. A stock solution of 100 mM of each compound in *N*,*N*-dimethylformamide (DMF) (<0.1%) was prepared prior to preparation of media solutions ranging from 0.05–100 μM. DMF was selected in place of DMSO as DMSO itself is known to be a free radical scavenger,^27^ thus limiting ROS within the cells. Fluorescence was read using bottom reading mode with an excitation wavelength of 485 nm and emission of 535 nm (Tecan Infinite F200). Validation of this assay was not done as part of this work and is acknowledged as a potential limitation.^28^

#### Volatile phase

**Scheme 1:**
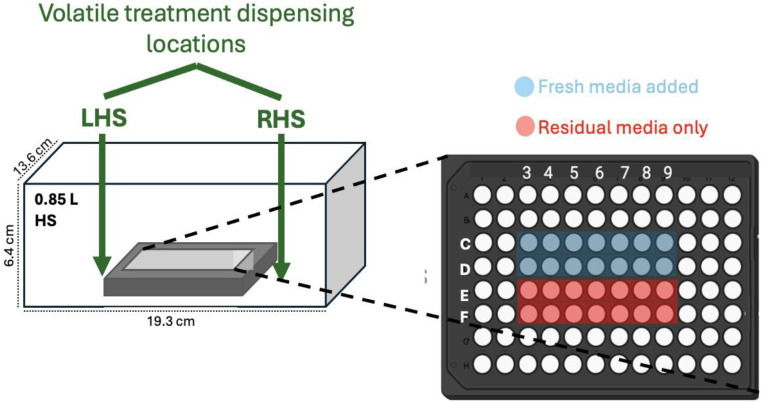
Experimental setup for volatile treatments of NHEK cell cultures for ROS assay. Left: 96-well plate housed in a glass HS chamber (0.85 L) within which, 0.5 μL volumes of neat compounds (nonanal, decanal, 6MHO, AA, or 2EH were dispensed as a liquid within a 1 cm distance of the midpoint of the LHS or RHS edge of the plate as specified (not to scale). Right: 96-well plate for ROS assay where cell-seeded wells C3–D9 have fresh media (20 μL) added and E3–F9 have residual media only.

Following incubation of plate (Rows C–F), media was aspirated from the wells and cells were prepared in one of two ways – (1) fresh media added (20 μL) or (2) left with residual media only, as specified. Plates were placed centrally in a glass headspace (HS) (0.85 L, dimensions: 19.3 × 13.6 × 6.7 cm, ‘Good For You’, https://www.amazon.co.uk) and the HS sealed using a lid (Scheme 1). Prior to closing, a neat volume of nonanal, decanal, 6MHO, AA or 2EH (0.5 μL) was then pipetted onto the bottom of the HS chamber within a 1 cm distance to the midpoint of the left hand side (LHS) or right hand side (RHS) edge of the plate (12.8 × 8.5 × 1.4 cm), as specified. The plate cover was then quickly removed and the lid of the chamber (snap-lock silicone seal) applied to close the HS. Assuming complete volatilisation of compounds dispensed into the chamber (Fig. 4a) and no sample loss, this would result in HS concentrations of ∼500 μg L^−1^. Exact HS concentrations assuming these idealized conditions based on compound densities are given in Table S1. The chamber was then placed in a static incubator at 37 °C for 2 h (CO_2_-free, homogeneous temperature distribution assumed).

In order to qualitatively validate the presence of the dispensed analytes in the HS after the 2 h incubation step, the HS of the chamber was sampled using solid phase microextraction (SPME) and analysed by gas chromatography-mass spectrometry (GC-MS). Experiments were performed in the absence (blank chamber) and with dispensed nonanal (as model analyte). Background VOCs recovered are also putatively identified and reported. All method information and data is presented in Table S2 validating that the chamber incubation conditions permitted volatilisation of the dispensed analytes and that the closed chamber retains nonanal whereby the concentration after 2 h incubation is significantly elevated relative to the blank chamber (>1500-fold).

Following incubation, the plate was removed from the chamber and all media aspirated from wells. Fresh media (100 μL) was added to wells followed by the ROS detection agent (100 μL). Cells were then incubated in 5% CO_2_ at 37 °C for 2 h. Fluorescence was read using bottom reading mode with an excitation wavelength of 485 nm and emission of 535 nm (Tecan Infinite F200).

### Immunofluorescence

#### Slide preparation

Cells were trypsinized and 200 μL of a 5 × 10^4^ cells per mL solution was seeded in glass bottomed 8-well plates (Ibidi, 80827) and allowed to grow in 5% CO_2_ at 37 °C for 24 h. Afterwards, the cell media was aspirated and cells treated with 200 μL volumes of with varying 1× concentrations (0.05–100 μM) of nonanal, decanal, 6MHO, AA or 2EH prepared in cell media for 5 h and 24 h.

#### Immunofluorescence staining

After 5 or 24 h as specified, media was removed from cells and cells washed three times using a wash buffer (phosphate buffered saline (PBS), 0.1% tween 20 (Sigma Aldrich, P1379) and 2% bovine serum albumin (BSA)). Cells were then fixed using 4% formaldehyde for 20 min, permeabilized with 0.5% Triton-X 100 for 45 min (Sigma Aldrich, Ireland) and blocked with 10% fetal bovine serum in PBS for 90 min. The cells were then incubated with primary rabbit-antihuman Nrf2 antibody (1 : 200) (ab62352, Abcam, Cambridge, UK) overnight at 4 °C. The next day the cells were further incubated in the primary antibody for 45 min at room temperature (RT). The primary antibody was removed and then cells were washed three times using the wash buffer solution. The cells were then incubated with a goat anti-rabbit IgG (H + L) cross-adsorbed secondary antibody, AlexaFluor 488 (1 : 1000) (Invitrogen, ThermoFisher Scientific, Ireland, A11008) for 1 h at RT in the dark. Secondary antibody was removed, and cells were washed 3× with wash buffer and then counterstained with DAPI (1 : 2500) for 3 min and then washed again with wash solution 3× before the addition of an antifade mountant.

#### Confocal imaging

Immunofluorescence was observed using a Leica DFC500 microscope equipped with a CCD camera and EBQ 100 power supply. AlexaFluor 488 was excited at 499 nm using a PicoQuant laser unit and the emission captured between 490 and 566 nm. DAPI was excited at 405 nm and emission recorded between 387 and 474 nm. Images taken using 20× magnification.

#### Statistical analysis

GraphPad Prism 10 software (La Jolla, CA) was used for statistical analysis. Each experiment was performed with at least 3 biological replicates and mean values ± standard deviation (SD) are reported. Significant differences were tested for using unpaired *t*-testing or one-way ANOVA analysis as specified.

## Results & discussion

### Cytotoxicity assay with skin-derived small metabolites

**Fig. 1:**
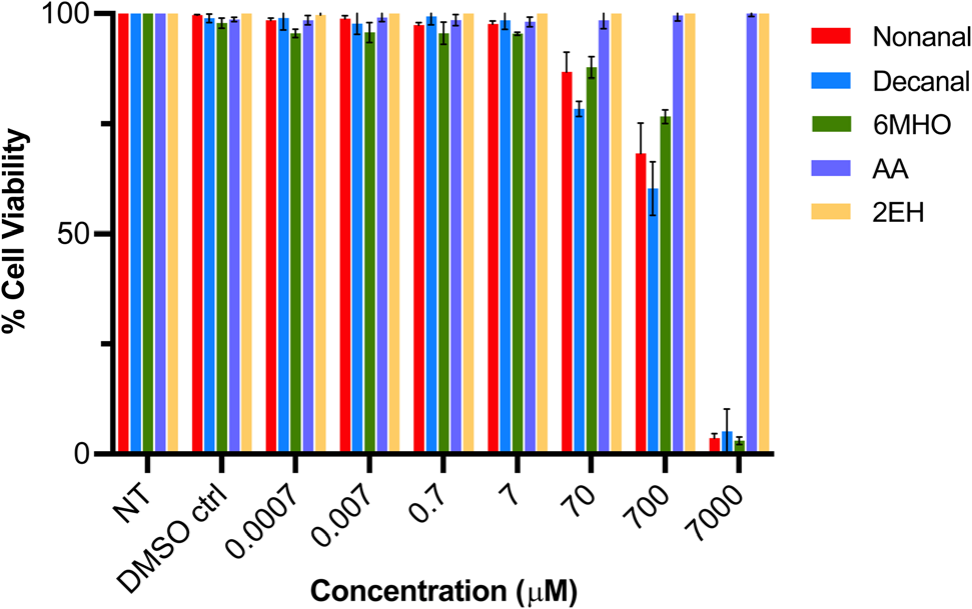
Viability of NHEK cells after 72 h treatment with different concentrations of nonanal, decanal, 6MHO, AA and 2EH (*n* = 3 biological replicates, *n* = 4 technical replicates), dissolved in DMF. Cells were incubated with PrestoBlue for 5 h following treatments. All values are the mean of *n* = 3 biological replicates ± SD. NT: non-treated.

Nonanal, decanal, 6MHO, AA and 2EH are small metabolites commonly reported to be present within the skin volatile profile^5,8^ and were selected for this study to evaluate their impact on signalling pathways in NHEK cells. The cytotoxicity towards NHEK cells of these metabolite compounds was first examined. Following assessment of the morphology of cultured NHEK cells (Fig. S1), the cytotoxicity of the compounds was investigated after exposure to the cells for 72 h. Cells were treated with specified concentrations of the different compounds (0.0007–7000 μM) and cytotoxicity determined, see Methods. % Cell viabilities after exposure to each of the compounds over the range of concentrations tested is shown in Fig. 1. No significant effect on cell viability was observed up to 7 μM for any compound. From 70 μM, cell viability was clearly reduced for nonanal, decanal and 6MHO. This effect continued at higher concentrations with >90% reduction in cell viability observed following exposure to 7000 μM of these compounds. Interestingly, no decrease in cell viability was noted for AA or 2EH, even at the higher concentrations tested. Based on these results, a concentration range of 0.05–100 μM was used for all compound treatments to investigate their full impact on NHEK cell signalling pathways.

### Triggering Nrf2 translocation with skin-derived small metabolites

**Fig. 2:**
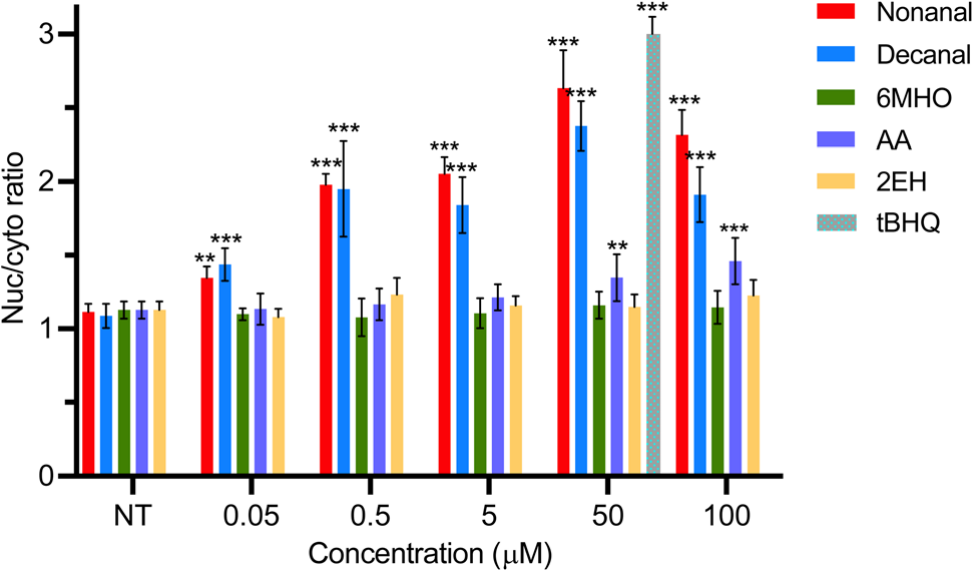
Nuc/cyto fluorescent intensity ratios from Nrf2 protein visualized by immunofluorescent staining after treatment for 5 h with nonanal, decanal, 6MHO, AA and 2EH in cell media. tBHQ (50 μM) served as the positive control. Ratios quantified using ImageJ. Immunofluorescent images available in Fig. S2. Values are the mean nuc/cyto ratio for *n* = 10 cells ± SEM. Stars above bar plots indicate a statistically significant difference in nuc/cyto ratio relative to their NT; * = *p* < 0.05, ** = *p* < 0.01, *** = *p* < 0.001).

To investigate if these metabolites of interest can trigger translocation of Nrf2 protein from the cytoplasm to the nucleus, NHEK cells were treated again with these compounds over the narrower concentration range 0.05–100 μM. Here, NHEK cells were stained with an Nrf2 antibody and fluorescent secondary antibody following treatments to investigate Nrf2 intracellular translocation (Fig. S2). The nuc/cyto ratio (signal intensity ratio of the cells' nucleus to cells' cytoplasm) was used to quantify nucleus translocation of protein (Fig. 2). When this ratio is <1, protein is predominately located in the cytoplasm indicating no translocation of protein, while a ratio of >1 indicates translocation of protein to the nucleus. A ratio =1 indicates protein is distributed equally across the nucleus and cytoplasm.

Initially, compounds in DMSO were added to cell media to specified concentrations and exposed to NHEK cells for 5 h as treatment. The non-treated (NT) control exhibited a nuc/cyto ratio of ∼1, indicating Nrf2 protein is evenly distributed across the nucleus and cytoplasm (Fig. 2). Translocation of Nrf2 protein was seen in cells following exposure to nonanal and decanal at concentrations as low as 0.05 μM (Fig. 2) and this finding is supported by previous work^21^ that examined activation of same pathway using aldehyde concentrations, albeit from higher concentrations (>2 μM).

At concentrations greater than 0.05 μM, concentration-dependent translocation was observed for both aldehydes up to 50 μM (Fig. 2) where there was no significant difference in ratios between the two treatments (*p* = 0.23; 50 μM). Treatment at 100 μM for either aldehyde did not lead to any further increase, potentially linked to the cytotoxic effect observed earlier (Fig. 1) at these concentrations. In terms of treatment time, no further translocation of Nrf2 protein was observed when cells were treated for up to 24 h with either nonanal or decanal (Fig. S3) indicating that the mechanism by which translocation is activated had largely ceased by 5 h, and Keap1, the negative regulator of Nrf2, has sequestered Nrf2 protein back in the cytoplasm.

AA also induced translocation, demonstrated by a significant increase in nuc/cyto ratio, albeit only at the highest concentration investigated (100 μM). AA, recovered as a microbial metabolite from the HS of human skin colonising *S. aureus* and *S. epidermidis* bacteria cultures^29^ is interesting as these bacteria are considered to potentially play a role in Nrf2–Keap1 pathway activation in skin^30^ but further experimentation would be needed to determine if AA plays a specific role in this. It is worth noting that AA is miscible with water and exhibits a significantly higher vapour pressure than the aldehydes. It is shown to undergoes translocation, albeit to a significantly lesser extent than the aldehydes. It may be that this lower level of translocation is driven by it's lack of lipophilic character compared to the aldehydes.

No translocation was observed for either 6MHO or 2EH (Fig. 2 and S2d, e)) indicating that neither compound plays a role in cellular defence against oxidative stress. These compounds have lower lipophilicities potentially lessening their ability to cross the cell membrane. Also, given the likely exogenous sources of these compounds, as discussed earlier,^9^ it is not surprising that they do not play a role in this regard.

### Elucidation of the mechanism of Nrf2 induction

#### Concentration dependent ROS generation induced by skin metabolites

Following confirmation of Nrf2 translocation upon treatment with nonanal, decanal and AA, the mechanism by which this translocation is activated was considered. Translocation is associated with an increase in mitochondrial cell membrane potential.^31^ An increased mitochondrial cell membrane potential was demonstrated previously upon cell treatment with aldehydes nonanal, decanal, benzaldehyde and 3-furaldehyde, and a mechanism of indirect pathway activation proposed on account of the increased ROS production.^21^

**Fig. 3:**
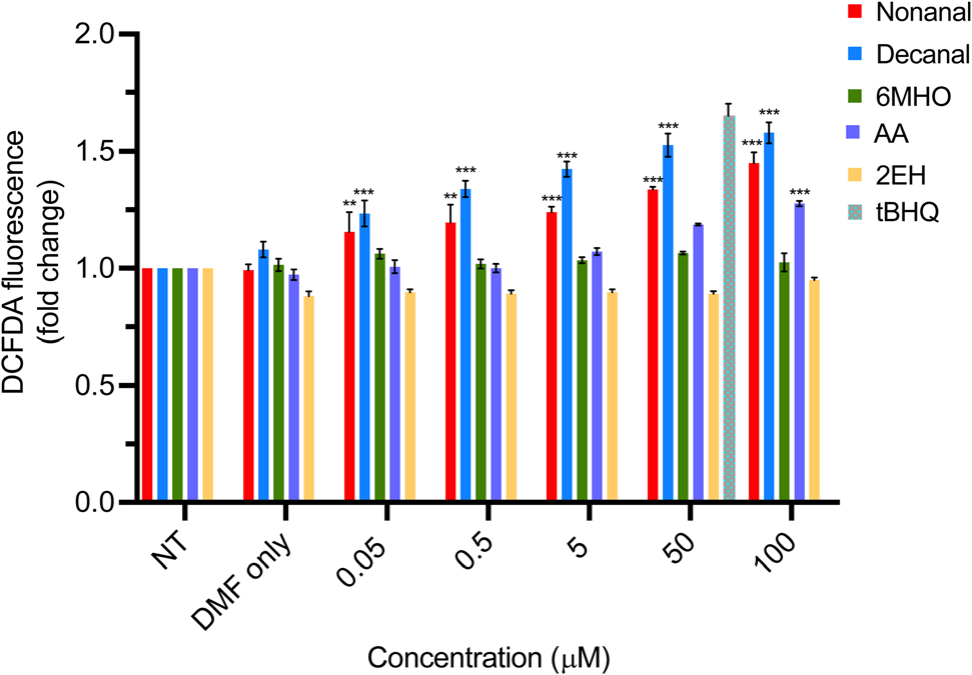
Endogenous ROS production after treatment of NHEK cells with nonanal, decanal, 6MHO, AA, 2EH for 2 h. tBHQ (50 μM) used as control. All values are the mean of *n* = 3 biological replicates ± SD. Stars above bar plots indicate a statistically significant difference in fold change relative to NT; * = *p* < 0.05, ** = *p* < 0.01, *** = *p* < 0.001.

To investigate ROS generation in our work, ROS production by NHEK cells was monitored following compound treatments using a positive Nrf2 inducer as control,^32^ see Methods. This allowed the probing of the mechanism by which the Nrf2-Keap1 cellular defence mechanism can be induced. ROS generation in response to nonanal, decanal, 6MHO, AA, 2EH (0.05–100 μM) was monitored following 2 h treatments (Fig. 3). Exposure to both nonanal and decanal showed significant elevation in ROS across all concentrations tested. Interestingly, ROS generation was higher for decanal than nonanal, relating potentially to its increased lipophilicity and hence enhanced ability to cross biological membranes.^33^

ROS generation was also observed for AA, albeit only at higher concentrations (Fig. 3), indicating that the activation of the Nrf2 pathway that was triggered at high concentrations (Fig. 2) can be linked to increased mitochondrial ROS production. Interestingly, AA, as a by-product of microbial metabolism, is recovered from skin in significantly higher abundances than the other compounds (except for 2EH) selected for study here.^9^ This is likely explained by metabolising microbes reaching large numbers in regions on skin containing high densities of sweat glands and pilosebaceous units (up to 10^6^ CFU cm^−3^)^34^ and hence it is speculated that products such as AA could potentially be reaching the high concentrations needed to trigger pathway activation. Further studies would be needed to quantify AA skin fluxes to understand how relevant these *in vitro* experiments might be to the *in vivo* case.

Cells treated with nonanal and decanal, as compounds generating the highest ROS production, were re-tested after 24 h under the same conditions (Fig. S4) and the rate of ROS production was significantly lower at this timepoint compared to 2 h (Fig. 3), likely linked to the instability of ROS over time within the cell environment.

Exposure to 6MHO showed no significant ROS elevation at any concentration. ROS production was also not observed for 2EH, again consistent with the absence of translocation. Both these compounds have lower lipophilicities than the aldehydes. The increase in ROS production within the cells upon treatment with the other compounds suggests that the mechanism by which the Nrf2-Keap1 pathway is activated indirectly is due to an augmentation of ROS. It could be hypothesized that nonanal, decanal and potentially also AA provide an adaptive response, in a similar way to HNE, albeit by an indirect mechanism. This causes the production of phase II enzymes following translocation of Nrf2 to the nucleus which provide cells with protection against further oxidative stress.

### ROS generation induced *via* gas phase diffusion of skin metabolites

Research has shown that microbial volatiles have roles in distant interactions and communication with other microbes as well as hosts.^35^ Volatile-mediated interactions in hosts^36^ and between microbes and hosts^37^ have been elucidated in both the plant and animal kingdoms, having a role for example in alerting host communities to local environmental stress exposures. These small volatile compounds are the first microbial signal to reach a target organism as they can diffuse rapidly in the gas phase and also diffuse more rapidly in the liquid phase than their polar, non-volatile counterparts. However, the receptors and the regulatory genes and pathways involved in their recognition by the target organisms are still largely unknown. Thus it was investigated here if the skin metabolites shown here to trigger ROS production in keratinocyte cells, and believed to be at least in part microbially-derived, can trigger this same ROS response using volatile transmission to reach the cells.

**Fig. 4:**
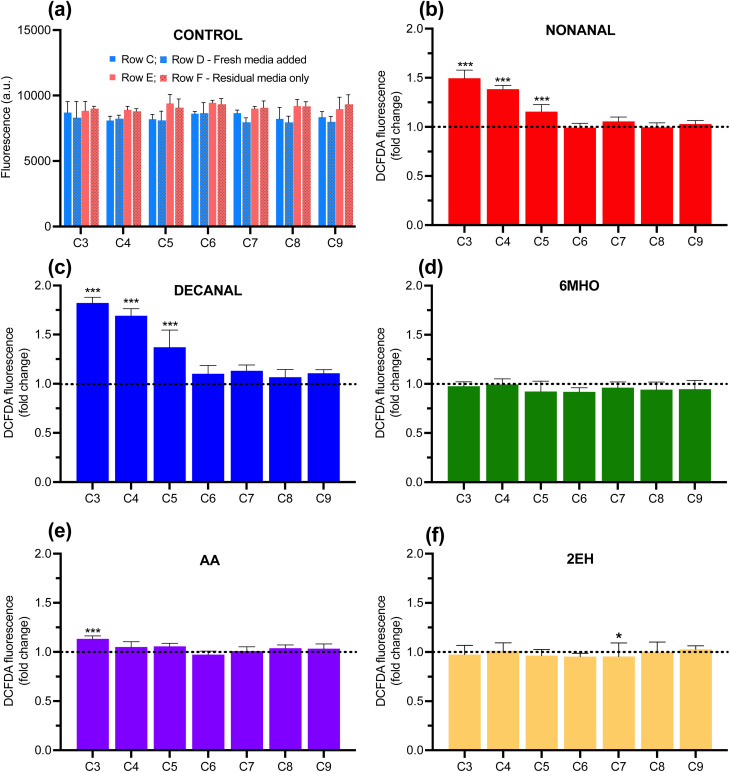
(a) ROS production along each column (C) after a 2 h incubation (no treatment), with rows C, D incubated with fresh media (blue) and rows E, F with residual media only (red). Values are the mean of *n* = 3 biological replicates ± SD. (b–f) ROS production along each column for wells with residual media only after incubation with volatile compound treatments: (b) nonanal (c) decanal (d) 6MHO (e) AA (f) 2EH. All compounds dispensed (0.5 μL) to LHS of plate and incubated for 2 h at 37 °C in HS. Fig. S5 shows the ROS production when compounds dispensed to RHS under same conditions. Fig. S6 and S7 shows the ROS production in the presence of freshly added media when compounds dispensed to LHS and RHS, respectively, under same conditions. All values are the mean of *n* = 3 biological replicates from matched column wells (C3–C9) across duplicate rows (*n* = 6) ± SD. Stars above bar plots indicate a statistically significant difference in fold change relative to matched wells on the control plate; * = *p* < 0.05, ** = *p* < 0.01, *** = *p* < 0.001.

To investigate this, an experimental setup was designed where a seeded plate was prepared with wells having fresh media added or left with residual medial only (see Methods, Scheme 1) and incubated in the HS chamber (without plate lid) at 37 °C for 2 h. For treatments, neat volumes of compounds were dispensed to either side of a seeded plate (LHS or RHS, as specified) prior to incubation. Based on the volume dispensed, a maximum HS concentration of ∼500 ppb of compound was assumed based on HS volume. For control plates, no compound was dispensed. Following the 2 h incubation, the production of ROS for each well was measured (Fig. 4). The control plates (Fig. 4a) allows us quantify if there are non-uniform media evaporation effects driving ROS production in cells incubated with residual media only. We observe a small, uniform increase in ROS production for all columns with wells with residual media only relative to those with fresh media added (Fig. 4a). This shows us that starting the incubation with residual media only systematically results in an increase in ROS (<15% in all cases) due to evaporation effects, but importantly, that this effect is uniform over the plate.

In this case of incubations with volatile phases of the compounds, ROS production is shown for plate columns (C) in Fig. 4b–f, where residual media only was in wells (no added media) and the compound treatment was dispensed to the LHS of the plate (Scheme 1). The ROS response is reported as relative to the matched control well fluorescence response (Fig. 4a). Significant ROS production was observed for nonanal, decanal and AA in the outer left column. Interestingly, moving across the plate, a gradient decay in response was observed. This trend was mirrored when compound treatments were dispensed to the RHS of the plate (Fig. S5). Thus, for compounds expected to trigger ROS production, a gradient profile of ROS production is seen across the plate, dependent on treatment dispensing location. This can be explained by a combination of effects whereby gradient-style evaporation is occurring from the outer to the inner wells, which in itself does not cause an ROS response (Fig. 4a), but, results in cells in the outer peripheral wells being most directly accessible *via* the gas phase. This, in combination with differing HS spatiotemporal distributions for each volatile, dependent on physicochemical properties (Table S3) during incubation, is likely driving the column-dependent ROS responses across the plate.

In terms of magnitude of response for each treatment, the highest production of ROS in the outer left column upon volatile treatment was observed for decanal (Fig. 4c), followed by nonanal (Fig. 4b) and AA (Fig. 4d), again consistent with that observed for cell media phase treatment using DMSO as carrier (Fig. 3). This is strong evidence that the gas phase of these compounds can trigger ROS generation from a long range distance, undergoing either a gas phase diffusion process to reach the cells directly in the absence of media, or, in the presence of cell media, a gas phase diffusion followed by partitioning into media and undergoing liquid phase diffusion, or a combination of both. However, given that nonanal and decanal have no solubility in water and DMSO was not used as carrier, it is likely that aqueous partitioning and liquid phase diffusion was not significant for these compounds. AA on the other hand, is water-miscible and therefore it is plausible that liquid phase diffusion processes could play a role in the mass transfer of AA to the cells in media. However, with the ROS response from AA being much lower than that of the aldehydes even directly from cell media using carrier (Fig. 3), it makes it difficult to fully conclude on the precise nature of the thermodynamics involved based on the data presented. Little or no production of ROS was observed for volatile 6MHO or 2EH treatments, consistent with the liquid phase treatments.

In order to ascertain the potential impact of media evaporation on the ROS production for the volatile phases of the different treatments (LHS and RHS), ROS production from column wells with fresh media added before incubation (Scheme 1, Rows C, D) was compared to the column wells with residual media only (Scheme 1, Rows A, B). A similar pattern of response was observed for both sets of wells, albeit a reduced response was noted for wells with fresh media (Fig. S6 and S7). The significance of fresh media compared to residual media was tested (Table 1) and was found to be significant for both outer columns in the case of nonanal, and only in outer columns closest to the dispensing location (LHS or RHS) for decanal. This difference may be explained by the differences in volatility and diffusion properties of these treatments, and thus, upon dispensing, assuming environmental conditions are equivalent for all experiments, treatment-specific spatiotemporal concentration distributions will arise in the HS during the 2 h incubation. In the case of nonanal, which exhibits higher vapour pressure and diffusivity than decanal, allows it be more rapidly transported across the HS to interact with cells in the outer wells furthest from dispensing point where media undergoes greatest evaporation. Decanal on the other hand is less volatile and will transport more slowly due to its heavier molecular weight and thus the resulting spatiotemporal distribution across the HS may prohibit the decanal reaching the outer wells, furthest from the point of dispensing. The finding that there was no significance for the inner wells in this analysis for either aldehyde indicates that ROS generation was impeded significantly in the presence of any volume of aqueous media, and thus supports the hypothesis that significant partitioning and diffusion of these aldehydes in the media is unlikely.

**Table 1:** Significance testing for the mean ROS production between cells with freshly added media (*n* = 6) (LHS data in Fig. S6; RHS data in Fig. S7) and cells with residual media only (*n* = 6) (LHS data in Fig. 4; RHS data in Fig. S5) for each of the volatile phase treatments nonanal, decanal and AA. Unpaired *t*-testing applied per column × 3 plates. ns = non-significant, * = *p* < 0.05, ** = *p* < 0.01, *** = *p* < 0.001

Volatile HS treatment	Dispensing to LHS	C3	C4	C5	C6	C7	C8	C9	Dispensing to RHS
Nonanal	√	***	***	ns	ns	ns	**	***	
		ns	**	ns	ns	ns	***	**	√
Decanal	√	***	***	ns	ns	ns	ns	ns	
		ns	ns	ns	ns	ns	***	**	√
AA	√	***	ns	ns	*	ns	ns	ns	
		ns	ns	ns	ns	ns	ns	*	√

In the case of AA, the response difference between wells with freshly added media and residual media only was less significant overall (Table 1), albeit it does indicate that cell media did impede mass transfer but potentially not as significantly as for the water-insoluble aldehydes.

These effects discussed support the hypothesis that cell media evaporation and compound-specific volatilisation and diffusion processes, and their interaction, are playing a role in the ROS responses observed. The results show that the ROS response can be triggered directly from the gas phase, although some aqueous phase partitioning and liquid phase transport may play a role, for AA in particular. Non-uniform water evaporation across the plate and VOC-specific spatiotemporal distributions during incubation are likely influencing the degree of interaction of the compounds with the cells, yielding specific ROS response profiles for the different compounds.

These experimental results provide evidence that nonanal, decanal and AA produced in skin can directly trigger ROS generation from a distance *via* gas phase interactions with keratinocyte cells. The magnitude of this gas phase interactive effect with cells is dependent on the nature of the volatile and its physicochemical properties, is dose dependent, and is dependent on the aqueous content surrounding the cells. It provides an initial proof of concept that specific skin-emitted VOCs can drive ROS production directly from the volatile phase, which can lead to the activation of the Nrf2-Keap1 protective pathway in cells.

## Conclusion

This study probes a largely uncharacterized role for skin-emitted VOCs in modulating intracellular redox signalling pathways in human keratinocytes. We demonstrate that selected skin-emitted volatiles – nonanal, decanal, AA – trigger ROS generation and activate the Nrf2–Keap1 antioxidant response pathway from both liquid and gas-phase exposure. The gas-phase activation represents a potential mechanism of cellular communication within the skin microenvironment that is driven by short-range diffusion of volatile metabolites. These findings highlight the potential for a chemical signalling function for endogenous and microbially-derived volatiles beyond their established roles in diagnostics or olfaction. This work provides a mechanistic foundation for exploring VOCs as gaseous modulators of skin homeostasis and opens new avenues for investigating volatile-phase signalling in other biological systems. Future studies will investigate more complex systems including testing a broader range of VOCs and mixtures thereof, assessing redox signalling pathways directly from microbially-derived volatile emissions, as well as considering air–liquid interfaces or organ-on-a-chip models to provide further insights into the physiological relevance of this proposed mode of cell–cell interaction.

## Conflicts of interest

Nothing to declare.

## Supplementary Material

RA-015-D5RA02839F-s001

## Data Availability

The datasets supporting the findings of this study, including raw fluorescence values, ROS assay outputs, and immunofluorescence image files, are available from the corresponding author upon reasonable request. Supplementary information is available. See DOI: https://doi.org/10.1039/d5ra02839f.
