# Supplementary material for: Skin-derived volatile organic compounds trigger redox signalling pathways in human keratinocytes via gas-phase interaction
Source: RSC Adv. 2025 Sep 10;15(39):32768–77. doi: 10.1039/d5ra02839f (PMC12421873; doi:10.1039/d5ra02839f)
Supplement: RA-015-D5RA02839F-s001 [file RA-015-D5RA02839F-s001.pdf]

Supplementary Information (SI) for RSC Advances.
This journal is © The Royal Society of Chemistry 2025

Supplementary Information

Skin-derived volatile organic compounds trigger redox signalling pathways in human keratinocytes via gas-phase interaction

Finnegan, M.[1], Bolikava, V. [1], Walsh, N.[2], Morrin, A.[1]

[1]School of Chemical Sciences, Insight Research Ireland Centre for Data Analytics, Dublin City University, Ireland, D09 V209
[2]School of Biotechnology, Life Sciences Institute, Dublin City University, Ireland, D09 V209

(a)

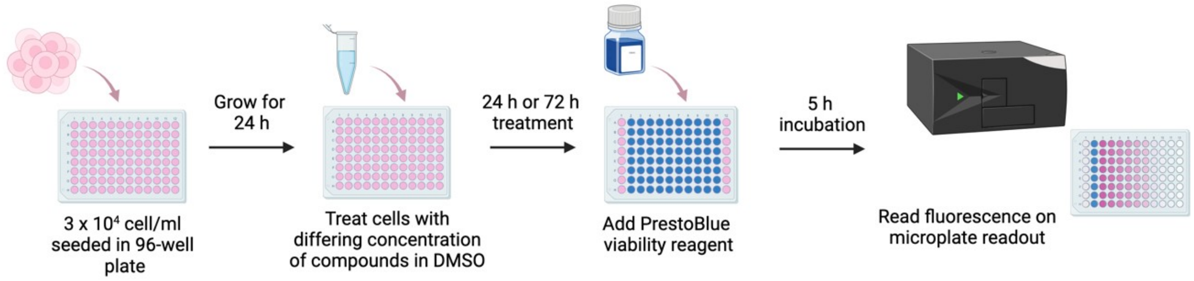

(b)

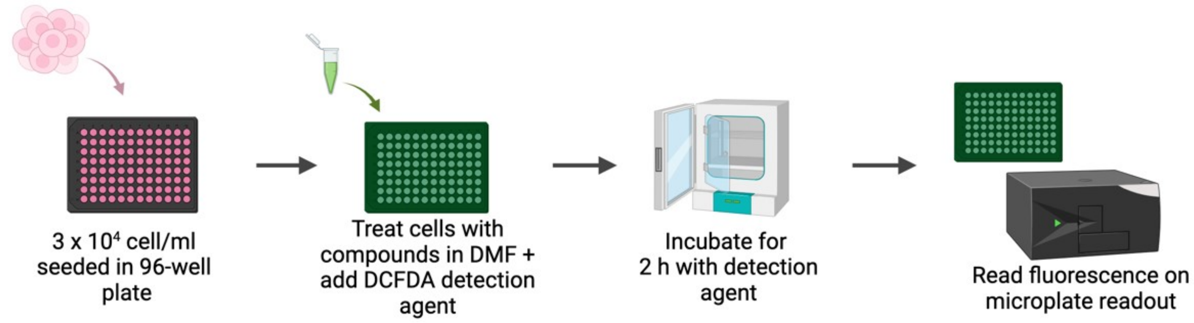

Scheme S1: **(a)** Protocol for the cell cytotoxicity assay and (b) protocol used for ROS assay in liquid phase.

Table S1: Theoretical HS concentrations of VOCs assuming complete vaporisation in HS and no VOC loss over the duration of the 2 h incubation of cells with dispensed compounds.

**Compound**	**Density (g/mL)**	**Mass of 0.5 μl (mg)**	**Theoretical HS concentration (mg/L)**
**Nonanal**	0.83	0.415	0.488
**Decanal**	0.83	0.415	0.488
**6MHO**	0.86	0.429	0.505
**AA**	1.04	0.520	0.612
**2EH**	0.83	0.416	0.490

Table S2: Retention times (RT), compound identities and recovered peak areas for compounds detected in the HS of the blank chamber and chamber with dispensed nonanal (0.5 μL) after incubation for 2 h at 37°C.
The chamber HS was sampled for VOCs using solid phase microextraction (HS-SPME) gas chromatography-mass spectrometry (GC-MS) using a standard method with technical specifications provided in [1]. Compounds listed were putatively identified by comparison with NIST2017 database where possible. All siloxane compounds recovered were excluded from table as are likely derived from SPME fibre.
Peak area mean ± standard deviation (n=3 unless otherwise specified) is reported. Average peak area for nonanal increased by >1500-fold, a significantly greater fold increase than other compounds.

**RT (min)**	**Compound identity**	**Average peak areas for compounds recovered from blank chamber (n=3)**	**Average peak areas recovered from incubated chamber containing 0.5 μL nonanal (n=3)**	**Fold differenc e**
**2.969**	n-Hexane	102797 (recovered from one sample only)	117968 (recovered from one sample only)	1.15
**9.702**	Oxime-, methoxy-phenyl-	1654469 (recovered from two samples only)	318084 ± 168773	0.19
**12.423**	2EH	83054 ± 54863	318084 ± 168763	3.83
13.763	***Nonanal***	***289916 ±204827***	***49479148 ±13179503***	***1706.67***

Morphology of the adult NHEK cells

Cell morphology was examined at different stages of confluency. These NHEK cells had a doubling time of 24 h and are guaranteed for 18 population doublings. Figure S1(a) shows NHEK cells 2 days after being thawed back at approx. 20-30% confluency. Cells were allowed to grow for a further 3 days before they reached 60-70% confluency and were sub-cultured (Figure S1(b)). These cells were sub-cultured at 60-70% confluency as they can become irreversibly contact-inhibited which may trigger cellular senescence if allowed to grow past 80% confluency.

(a)

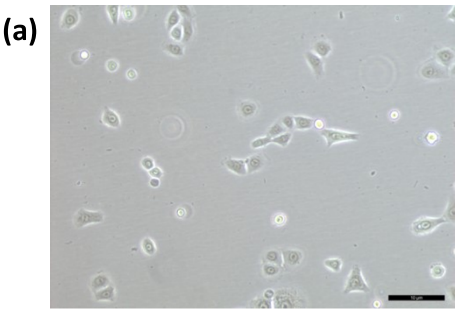

(b)

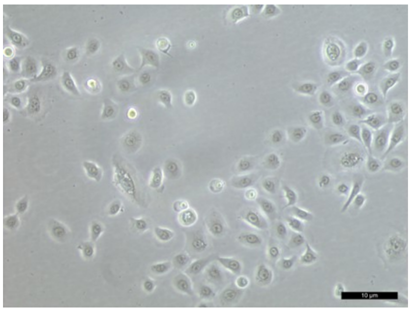

Figure S1. NHEK cell images at (a) 20-30% confluency and (b) 60-70% confluency. Images taken at 10X magnification. Scale bars = 100 μm.

(a) tBHQ

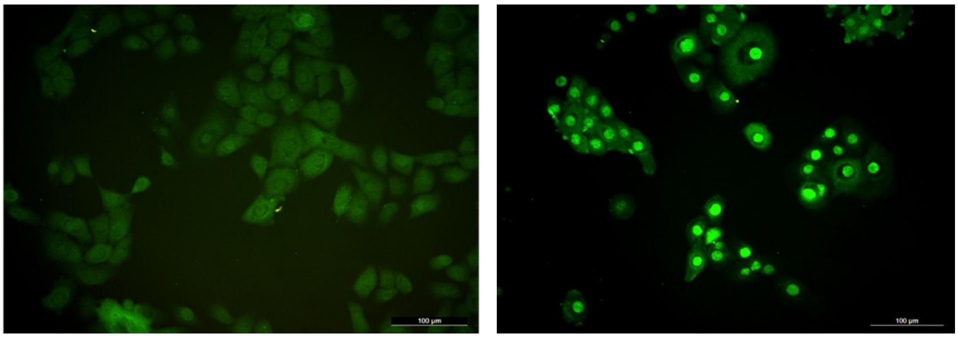

Control (No treatment)

50 ∝M

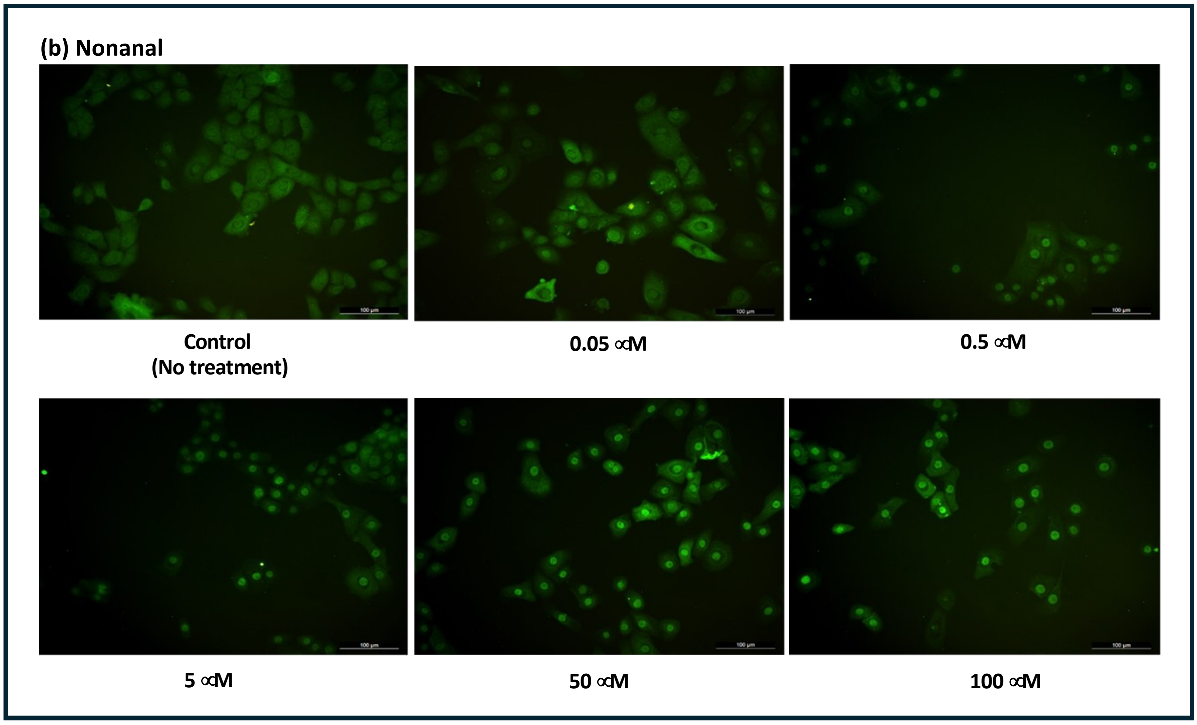
(b) Nonanal
Control
(No treatment)
0.05 ∝M
0.5 ∝M
5 ∝M
50 ∝M
100 ∝M

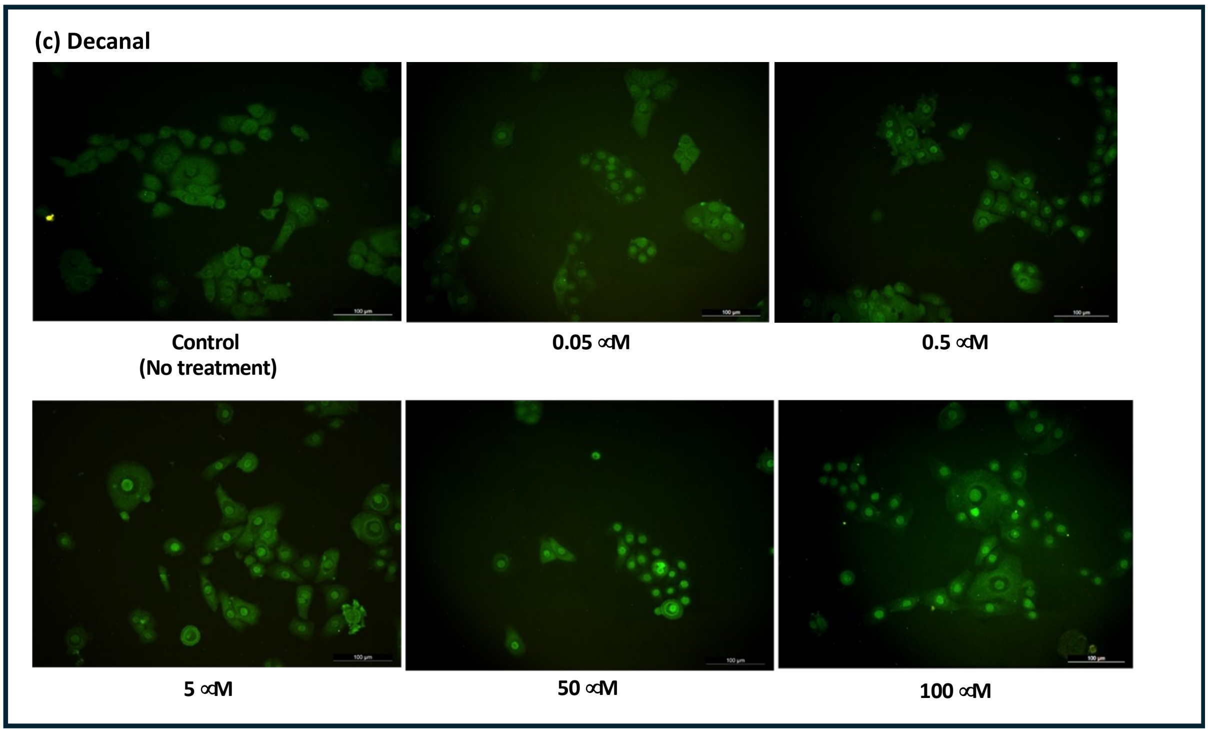
(c) Decanal
Control
(No treatment)
0.05 ∝M
0.5 ∝M
5 ∝M
50 ∝M
100 ∝M

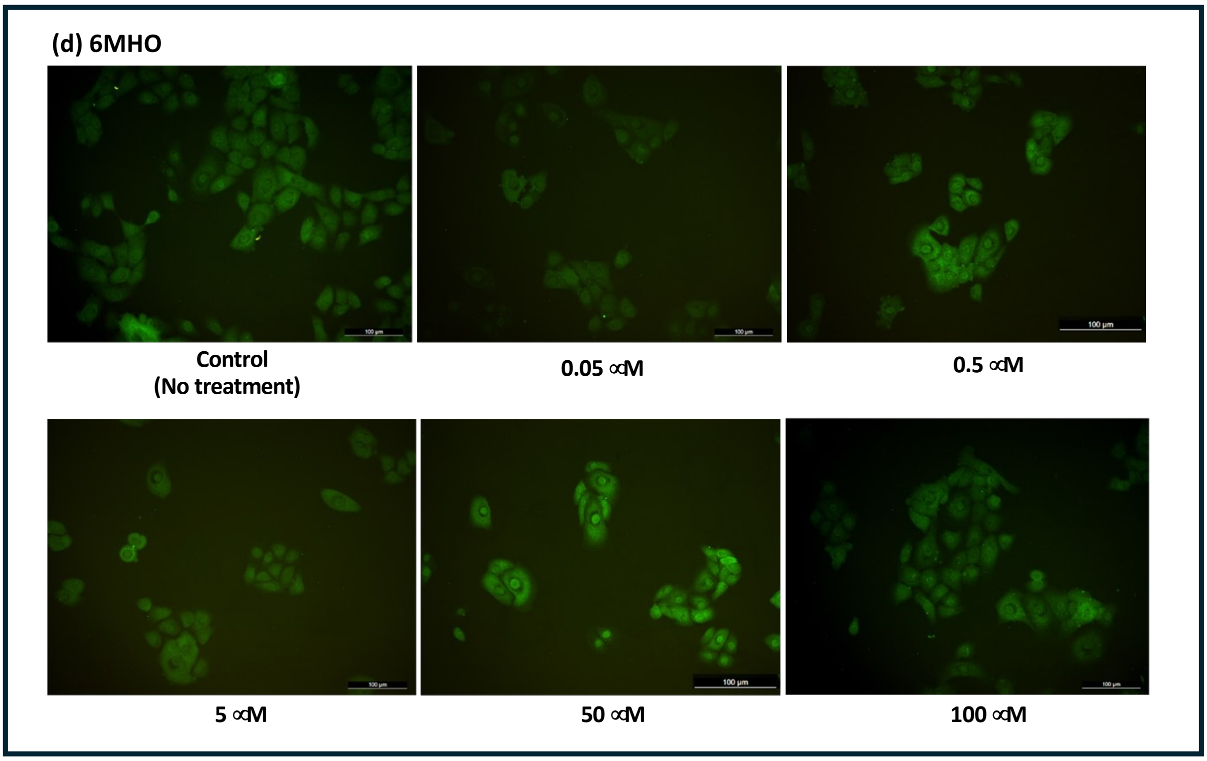
(d) 6MHO
Control
(No treatment)
0.05 ∝M
0.5 ∝M
5 ∝M
50 ∝M
100 ∝M

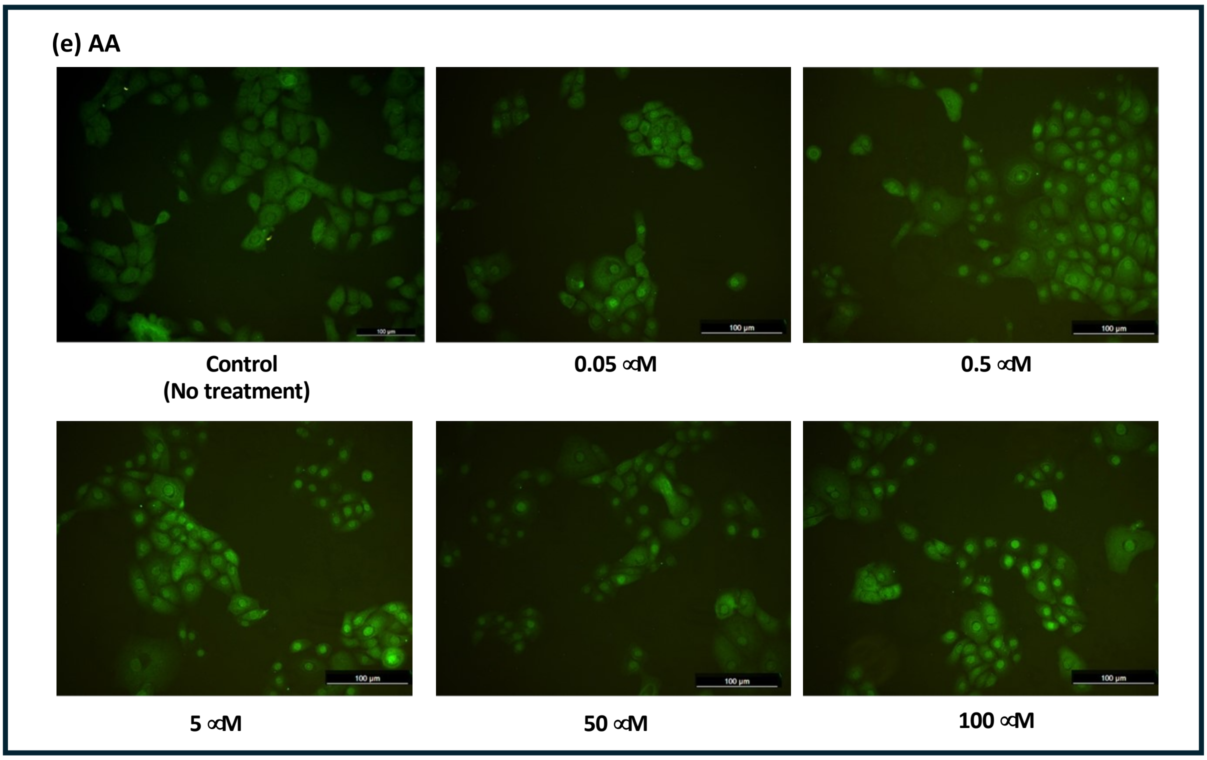
(e) AA
Control
(No treatment)
0.05 ∝M
0.5 ∝M
5 ∝M
50 ∝M
100 ∝M

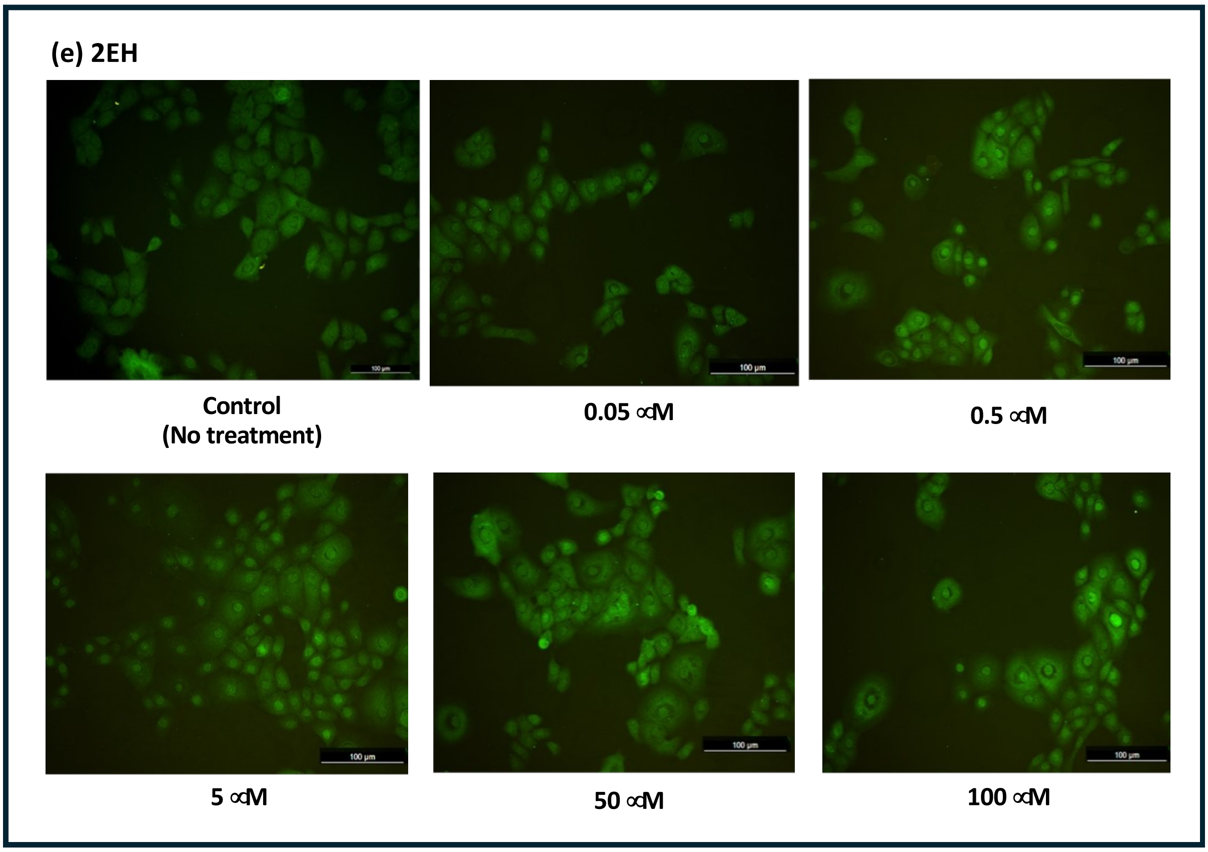

Figure S2. Nuclear translocation of Nrf2 protein visualised by immunofluorescent staining after treatment for 5 h with (a) 50 μM tBHQ in cell media and specified concentrations of (b) nonanal, (c) decanal, (d) 6MHO, (e) AA and (f) 2EH in cell media. Images taken at 20X magnification. Scale bars = 100 μm.

Table S3. Physicochemical properties of VOCs investigated in this study. All values are approximate and may vary slightly depending on measurement conditions and purity.

Property	Nonanal	Decanal	6-Methyl-5-hepten-2-one	Acetic Acid	2-Ethyl-1-hexanol
**Molecular Formula**	$C_9H_{18}O$	$C_{10}H_{20}O$	$C_8H_{14}O$	$C_2H_4O_2$	$C_8H_{18}O$
**Molecular Weight (g/mol)**	142.24	156.27	126.20	60.05	130.23
**Physical State at Room Temperature**	Liquid	Liquid	Liquid	Liquid	Liquid
**Boiling Point (°C)**	191	208-209	173-174	118	184-185
**Melting Point (°C)**	-19	-5	-67	16.6	-76
**Density (g/cm³, 20°C)**	0.827	0.830	0.846	1.049	0.833
**Vapour Pressure (mmHg, 25°C)**	0.3	0.1	1.2	15.7	0.05
**Solubility in Water**	Slightly soluble	Slightly soluble	Moderately soluble	Miscible	Slightly soluble
**Log P**	3.13	3.73	1.92	-0.17	2.73

**(octanol/water)**					
**Viscosity (cP, 20°C)**	1.7	2.3	0.9	1.22	9.8
**Surface Tension (mN/m, 20°C)**	27.4	28.0	25.2	27.6	27.5
**Dielectric Constant (20°C)**	8.1	7.8	13.6	6.2	7.6
**pKa**	N/A	N/A	N/A	4.76	~15
**Odor Description**	Fatty, citrus, waxy	Fatty, floral, waxy	Green, fruity	Pungent, vinegar	Mild, fatty

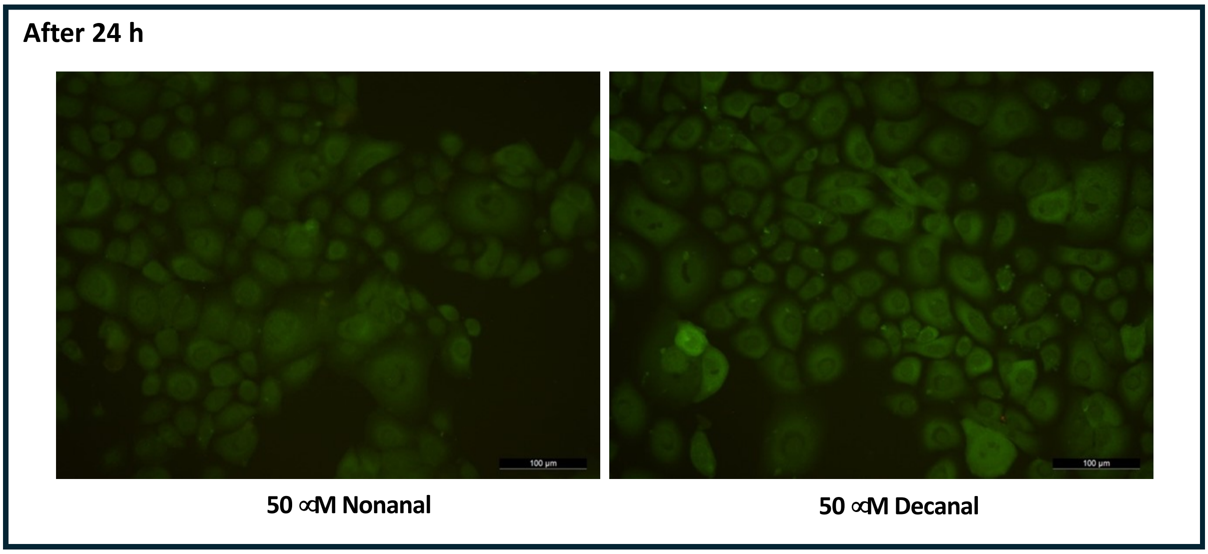

Figure S3. Nuclear translocation of Nrf2 protein visualised by immunofluorescent staining after treatment with nonanal and decanal (50 μM) in cell media for 24 h. (Images taken at 20X magnification. Scale bars = 100 μm).

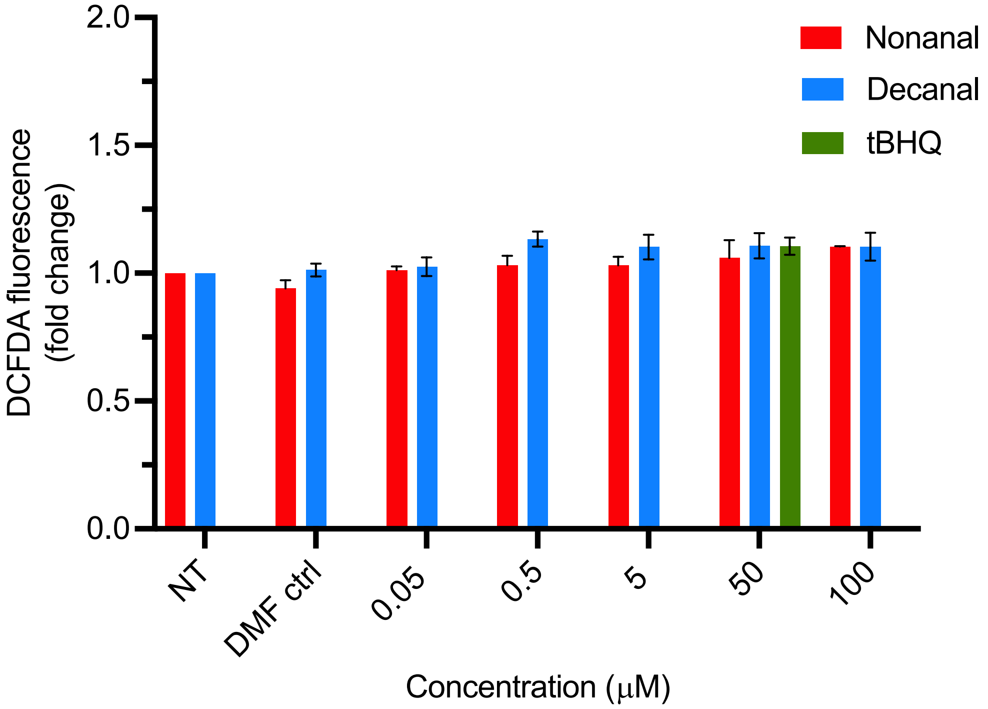

Figure S4: Endogenous ROS production after treatment of NHEK cells with nonanal and decanal in DMF for 24 h. tBHQ (50 μM only) used as control. All values shown are the mean of n=3 biological replicates ± SEM. Stars above bar plots indicate a statistically significant difference in fold change relative to NT; * = p<0.05, ** = p<0.01, *** = p<0.001).

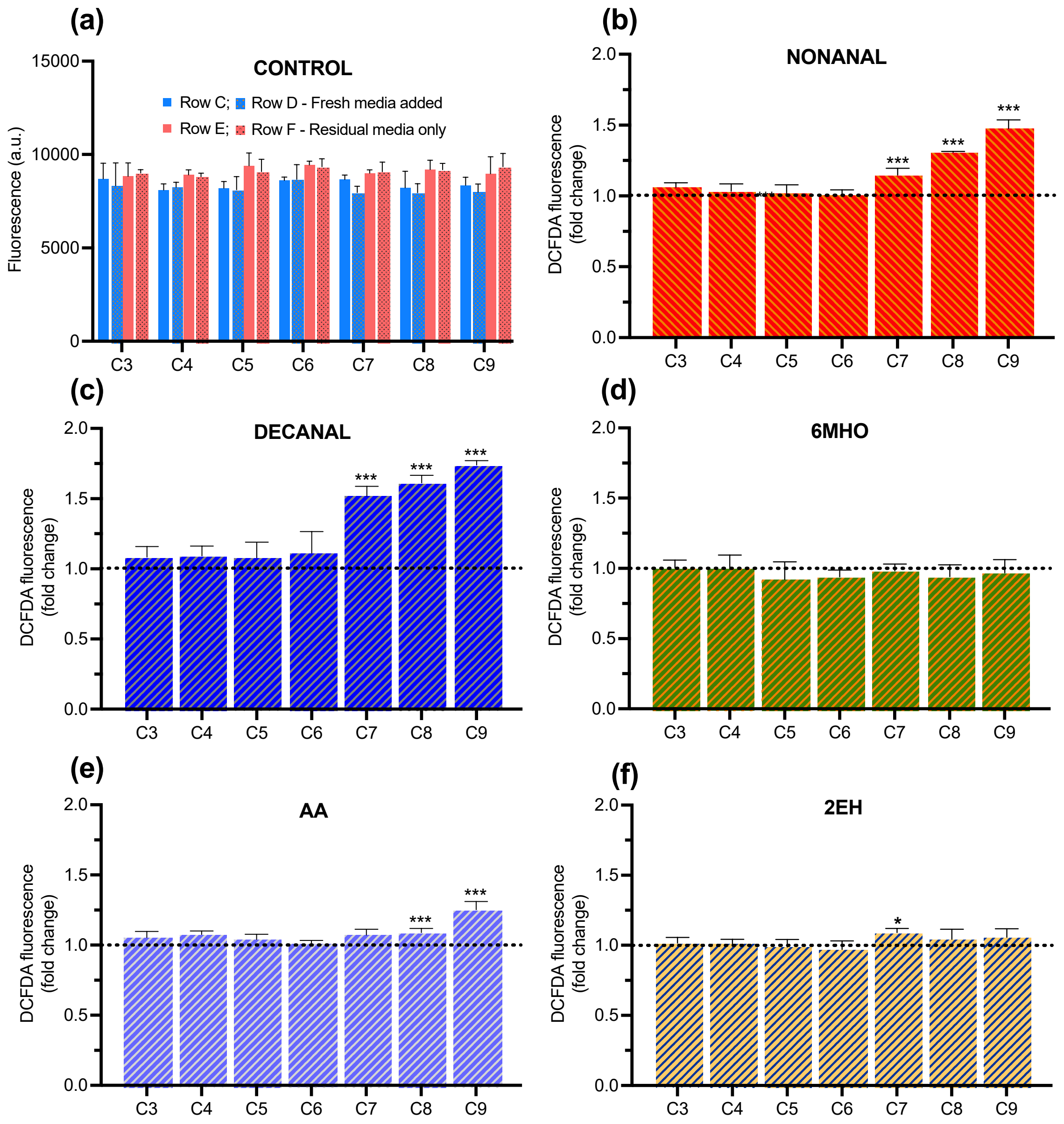

Figure S5. (a) ROS production along each column (C) after a 2 h incubation (no treatment), with rows C,D incubated with fresh media (blue) and rows E,F with **residual media** only (red). Values are the mean of n=3 biological replicates ± SD. (b-f) ROS production along each column for wells with residual media only after incubation with volatile compound treatments: (b) nonanal (c) decanal (d) 6MHO (e) AA (f) 2EH. All compounds dispensed (0.5 μL) to **RHS** of plate, incubated for 2 h at 37°C in HS. All values are the mean of n=3 biological replicates from matched column wells (C3-C9) across duplicate rows (n=6) ± SD. Stars above bar plots indicate a statistically significant difference in fold change relative to matched wells on the control plate; * = $p<0.05$, ** = $p<0.01$, *** = $p<0.001$.

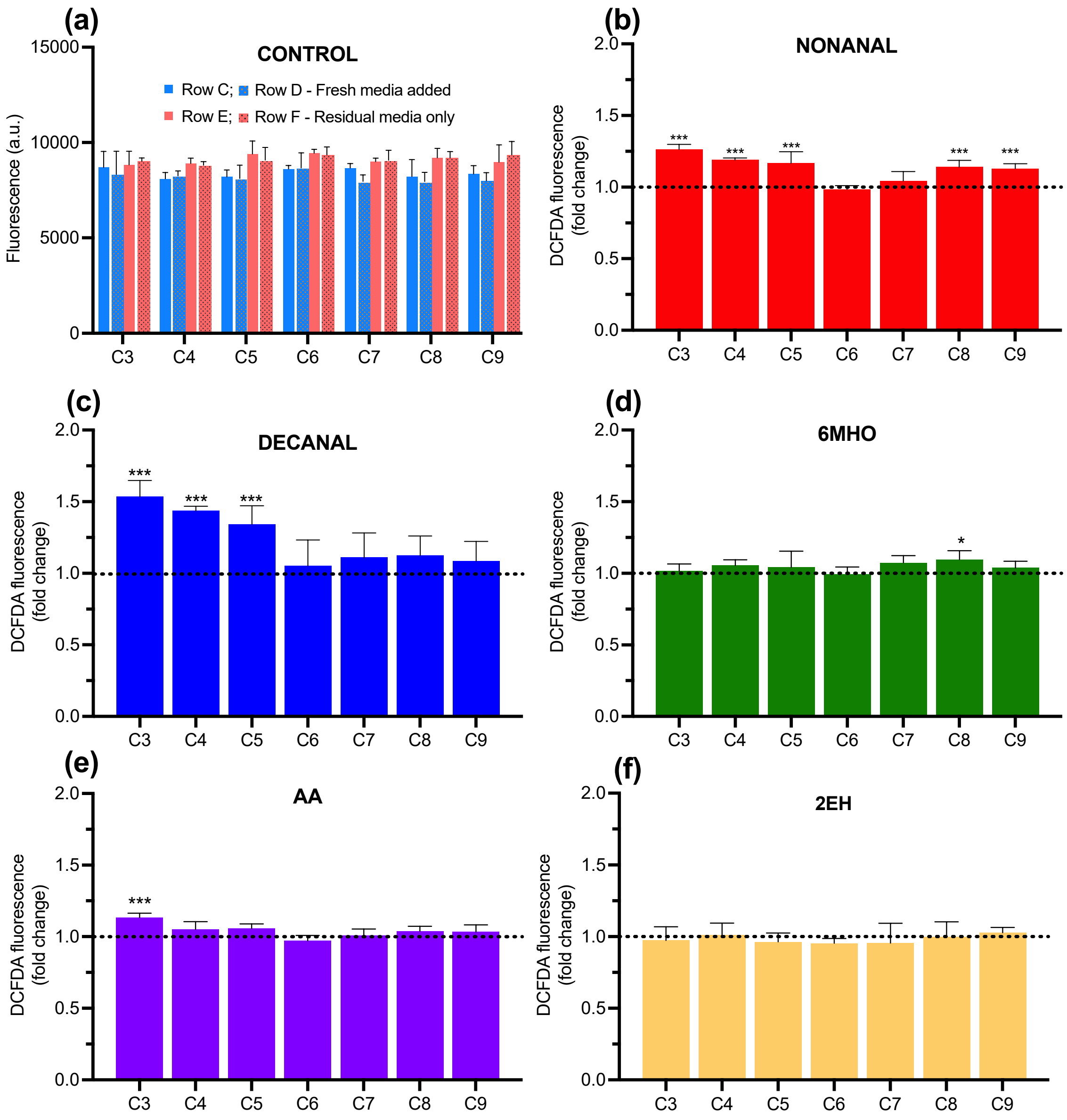

Figure S6. (a) ROS production along each column (C) after a 2 h incubation (no treatment), with rows C,D incubated with fresh media (blue) and rows E,F with **freshly added media** (red). Values are the mean of n=3 biological replicates ± SD. (b-f) ROS production along each column for wells with freshly added media after incubation with volatile compound treatments: (b) nonanal (c) decanal (d) 6MHO (e) AA (f) 2EH. All compounds dispensed (0.5 μL) to **LHS** of plate and incubated for 2 h at 37°C in HS. All values are the mean of n=3 biological replicates from matched column wells (C3-C9) across duplicate rows (n=6) ± SD. Stars above bar plots indicate a statistically significant difference in fold change relative to matched wells on the control plate; * = p<0.05, ** = p<0.01, *** = p<0.001.

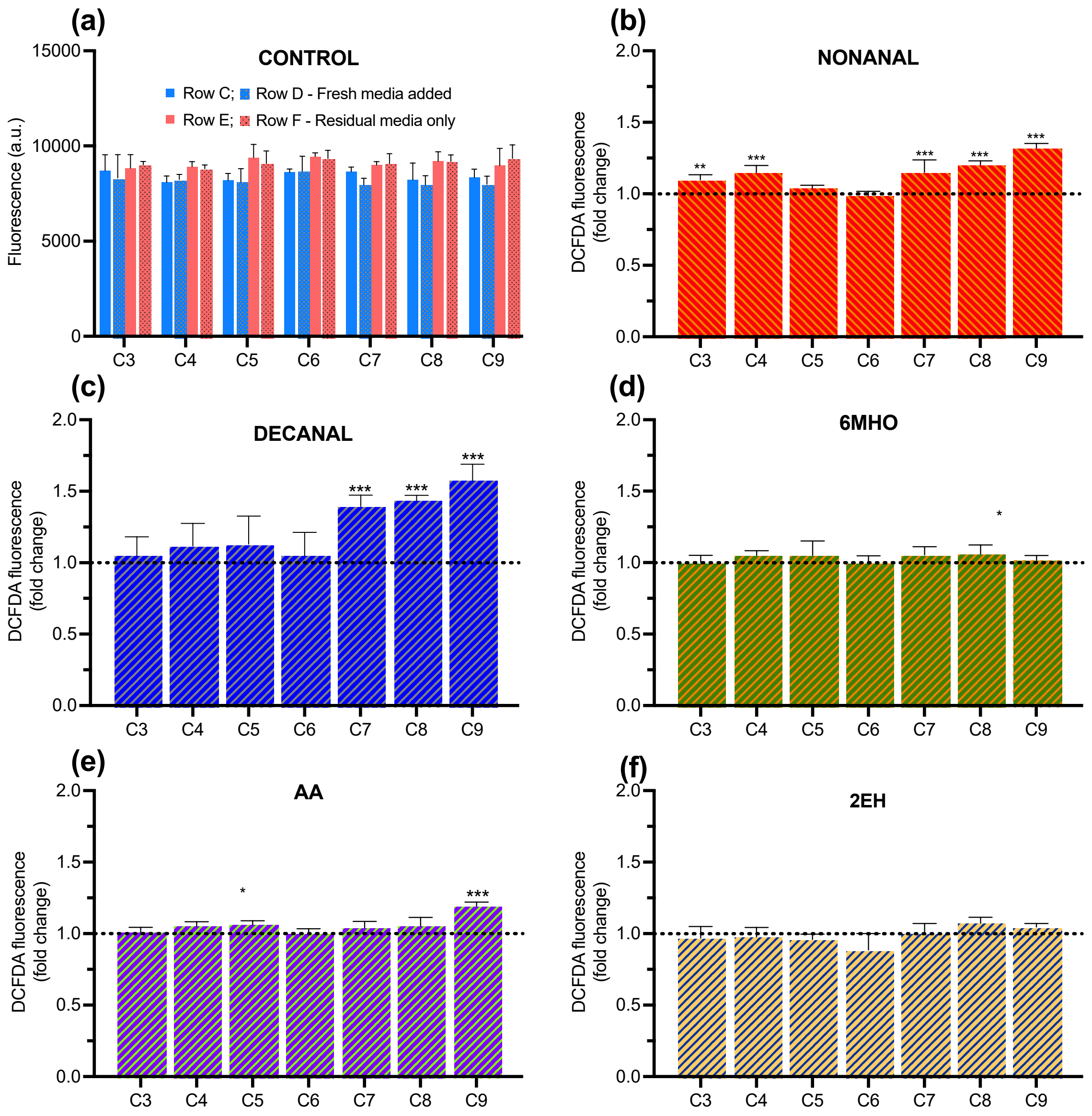

Figure S7. (a) ROS production along each column (C) after a 2 h incubation (no treatment), with rows C,D incubated with fresh media (blue) and rows E,F with **freshly added media** (red). Values are the mean of n=3 biological replicates ± SD. (b-f) ROS production along each column for wells with freshly added media after incubation with volatile compound treatments: (b) nonanal (c) decanal (d) 6MHO (e) AA (f) 2EH. All compounds dispensed (0.5 μL) to **RHS** of plate and incubated for 2 h at 37°C in HS. All values are the mean of n=3 biological replicates from matched column wells (C3-C9) across duplicate rows (n=6) ± SD. Stars above bar plots indicate a statistically significant difference in fold change relative to matched wells on the control plate; * = p<0.05, ** = p<0.01, *** = p<0.001.

References:

[1] Finnegan M, Fitzgerald S, Duroux R, Attia J, Markey E, O'Connor D, Morrin A (2024) Predicting Chronological Age via the Skin Volatile Profile. J Am Soc Mass Spectrom 35:421–432. https://doi.org/10.1021/jasms.3c00315
